# A Rural Tuberculosis Dispensary: A Guide to Its Equipment and Cost

**Published:** 1915-05-22

**Authors:** 


### A RURAL TUBERCULOSIS DISPENSARY.

### A Guide to its Equipment and Cost.

The desirability of establishing dispensaries at which patients are expected to attend is a debateable point in the case of entirely rural districts. In very sparsely populated areas the utility of such centres for treatment is quite incommensurate with their cost, and the loss of time suffered by the majority of patients in journeying to and fro is too serious a matter to expect them to be regular in their attendances. But in rural areas with a fair sprinkling of villages within a few miles of each other it may be well worth while to establish an inexpensive dispensary as a convenient treatment centre for a group of villages.

It will doubtless be objected that in any case the medical officer and nurse will have to pay frequent visits to the homes of patients under treatment, and that domiciliary supervision must, in the end, play the most important part in any scheme for the control of tuberculosis. Nevertheless, such a centre may serve many useful purposes. It may be utilised as a distributing base for such things as cod-liver oil, disinfectants, and spitting flasks. By appointment, several patients in the vicinity who are able to get about could be seen by the medical officer with a great saving of time; but, more usefully still, cases seen for the first time for the purpose of diagnosis could attend the dispensary and there be conveniently weighed and examined under better conditions than in their own homes. It must be borne in mind that a considerable number of cases notified to the medical officer of health are doubtful ones, and not infrequently the tuberculosis officer may come to the conclusion that a case does not need any treatment at the moment, and may content himself with making a careful record of the case for future revision. Such cases are preferably not visited in their homes if the suspicion of the existence of tuberculosis is not confirmed.

The "dispensary" may, of course, be installed in rooms in a house, but it will be found that in rural districts suitable accommodation of this description is not easily come by. A very satisfactory dispensary, such as the one about to be described, can be erected at a moderate cost on some vacant piece of land. The structure need not be a fixture, but one which with comparatively slight trouble and expense could be removed to another locality should administrative or demographic considerations favour such a change.

In describing a rural dispensary the writer has in mind one which has been installed in a village in the Midlands, but as regards the actual building a detailed specification is hardly called for, as the dimensions and structural arrangement will be varied according to the space available and its situation. To the details of equipment fuller consideration will be given, as these will, it is believed, form a useful guide as to what should be provided for a centre which is meant adequately to serve a rural district.

The building itself is a wooden structure about twenty-five feet long and ten feet broad, raised from the ground on short piers of bricks. The height of the walls is eight feet and the sloping roof is covered with a patent material called "Eternite." Guttering and down-spouting should be provided. There are six windows, four made to open casement fashion and two fixed ones. The building is divided into three unequal sized rooms and a short passage by three wooden partitions, the largest room at one end being the doctor's room, in which patients are examined and weighed; the smallest being the middle chamber, where patients can undress, and the third, intermediate in size, is used as a waiting room. The entrance door to the building is in the centre of one long side and opens into the small passage which is cut off from the depth of the middle room and connects through doors on either hand to the waiting room and the doctor's room respectively. On the other side of the structure, communication between these end rooms is established through the dressing room, there being two more doors in the transverse partitions, making four interior doors besides the entrance door. In the front of the building are two windows, one on either side of the entrance; at the back, only one window, lighting the dressing room; at the waiting room end, one window; and in the end wall of the consulting room, two windows, thus making three lights in this room. The two main rooms are warmed by gas fires, each provided with a flue leading to the eaves and passing directly outside through the wall behind the fire. Three incandescent gas brackets provide illumination for the interior; these are, however, rarely used, and it will be generally found that in the country it will be unnecessary to provide illumination except for laryngoscopic examinations, and for heating purposes oil lamps will perforce have to be used in most localities. If the building has to be used for longer time than an hour or two at a stretch, the problem of ventilation is apt to become acute, especially in the consulting room, and some opening in the roof will be called for in addition to the windows, especially during the winter months. The wooden floors are covered with linoleum and mats provided at the entrance.

As regards the furniture, the requirements of the two smaller rooms are simple. In the waiting room are placed five or six Windsor chairs and a few hooks for coats and hats. In the dressing room, which also serves as a dark room for examinations of the larynx, two chairs are required and some more hooks. A bracket for gas, or a support for an oil lamp or a portable electric light, and a shelf for instruments and spirit lamp are all that is necessary. The window must, of course, be provided with means for shutting out all light.

The consulting room is equipped with an examination couch covered with American cloth and having the head end raisable to any position; a desk-cupboard forming a writing table with a knee-hole on either side of which are stored disinfectants, tins of oil and malt, temperature charts, case papers, spitting flasks, etc.; a weighing machine; a small instrument cabinet; an aseptic dressing table; a cabinet with bandages and dressings; a japanned slop-pail; a steriliser with spirit lamps; a urine testing stand; a vertical filing cabinet and an office chair; a basin and jug on a tripod. With the exercise of a little ingenuity all these things can be so disposed in the room as to leave sufficient floor space free for the doctor and patient to move about. A few shelves on the walls will be found a great advantage.

The walls of the building both inside and out are painted a light colour. Such a structure with the fixtures, but without any equipment, can be built for about £60. It will be noted that no mention has been made of space and means for the bacteriological examination of the sputum. This is usually done at some central office in the county or perhaps in the residence of the tuberculosis officer.

In giving some details of the cost of apparatus and furniture it is only intended to show the expenditure in a particular case where there is no other centre for dispensary treatment and no chemist handy from whom cod-liver oil, etc., can be obtained; other medicines are procured from local practitioners.

| | £ | s. | d. |
|---|---|---|---|
| Examination Couch ... ... ... | 3 | 0 | 0 |
| Instrument Cabinet ... ... ... | 1 | 15 | 0 |
| Steriliser ... ... ... ... ... ... | 1 | 5 | 0 |
| Weighing Machine ... ... ... ... | 0 | 19 | 6 |
| Urine Test Stand ... ... ... ... | 0 | 10 | 0 |
| Dressing Table ... ... ... ... | 2 | 0 | 0 |
| Cabinet for Dressings ... ... ... | 1 | 0 | 0 |
| Basin and Jug and Tripod ... ... | 0 | 11 | 6 |
| Slop Pail ... ... ... ... ... ... | 0 | 3 | 9 |
| Vertical Filing Stand ... ... ... | 1 | 10 | 0 |
| Eight Windsor Chairs ... ... ... | 1 | 8 | 0 |
| Looking Glass ... ... ... ... | 0 | 2 | 3 |
| 500 Temperature Charts ... ... ... | 0 | 8 | 3 |
| One dozen Pulse Glasses ... ... ... | 0 | 6 | 6 |
| Four dozen Sputum Flasks ... ... | 1 | 0 | 0 |
| Six Urine Glasses ... ... ... ... | 0 | 4 | 0 |
| One Urinometer ... ... ... ... | 0 | 1 | 9 |
| One dozen Thermometers ... ... ... | 0 | 8 | 6 |
| One Laryngoscope ... ... ... ... | 1 | 6 | 0 |
| One Tuberculin Syringe ... ... ... | 0 | 8 | 6 |
| Quanti-Pirquet Outfit ... ... ... | 1 | 7 | 6 |
| Six Towels ... ... ... ... ... | 0 | 5 | 0 |
| Six Bowls ... ... ... ... ... ... | 0 | 2 | 3 |
| Six dozen Bottles in Boxes for Posting Sputum ... ... ... ... ... | 0 | 15 | 0 |
| 500 Carrier Paper Bags ... ... ... | 1 | 3 | 0 |
| Ten yards Flannelette for Patients' Jackets ... ... ... ... ... | 0 | 8 | 0 |
| One dozen Sputum Cups ... ... ... | 0 | 11 | 6 |
| One Desk Cupboard ... ... ... | 1 | 7 | 6 |
| One Office Chair ... ... ... ... | 0 | 10 | 6 |
| One gross 8-ounce Bottles of Cod-liver Oil | 2 | 6 | 0 |
| Three dozen Tins Oil and Malt ... ... | 0 | 15 | 0 |
| One gross 8-ounce Tins of Diluted Disinfectant ... ... ... ... | 1 | 7 | 0 |

In addition, there were purchased for use in patients' homes a portable weighing machine (cost £1 6s. 6d.), a travelling tuberculin case with another syringe (£2), and an electric head-lamp for laryngoscopic work (£2). The item of paper carrier bags requires a word of explanation; these are so inexpensive that the luxury may be condoned. The bags are similar to those now frequently presented to customers at large shops for the purpose of carrying away purchases. They are much appreciated by patients who have to carry away a bottle of oil, a tin of disinfectant, a sputum flask, a thermometer, and a postal sputum bottle or some of these presents.

The pulse glasses are for use by those cases who are asked to record their pulse rate as well as temperature on the charts. The money expended on flannelette for jackets is well spent; these jackets are simply made and are a great comfort to the patients while waiting for examination. If it is to be the rule that patients are to be weighed without their boots—a justifiable refinement—then a few pairs of cheap slippers should be provided. With regard to the tins of disinfectant, it will be noted that this is already diluted to the strength which is to be poured into the sputum cups. The disinfectant quoted is Kerol, and the very convenient tins only cost 2¼d. each, not an excessive concession to the mentality of the majority of rural patients.

